# Supplementary material for: Pattern Analysis of Serum Galectins-1, -3, and -9 in Breast Cancer
Source: Cancers (Basel). 2023 Jul 27;15(15):3809. doi: 10.3390/cancers15153809 (PMC10417135; doi:10.3390/cancers15153809)
Supplement: Supplementary file 1 [file cancers-15-03809-s001.zip › Supplementary Table S1.pdf]

Supplementary Materials

Tables

Table S1. Summary of Serum Galectin Values by Cancer Patient and Sample Characteristic

Characteristic	Galectin-1		Galectin-3		Galectin-9	
	N	Mean (SD), ng/mL	N	Mean (SD), ng/mL	N	Mean (SD), ng/mL
**Gender**						
Female	136	23.96 (9.22)	136	11.51 (5.86)	76	9.53 (4.54)
Undocumented	2	28.62 (7.98)	2	13.47 (6.87)	1	13.06 (nan)
**Race**						
Asian	3	20.82 (7.11)	3	6.88 (2.21)	2	7.47 (1.69)
Black, African American	21	25.73 (5.74)	21	13.20 (4.81)	11	9.24 (1.98)
Latino	3	19.87 (9.55)	3	11.69 (4.22)	2	6.85 (3.85)
Multi-Racial	2	23.70 (0.25)	2	15.04 (3.20)	2	11.29 (2.30)
White	108	23.81 (9.85)	108	11.21 (6.09)	59	9.69 (5.00)
Undocumented	1	34.26 (nan)	1	18.33 (nan)	1	13.06 (nan)
**Ethnicity**						
Non-Spanish; Non-Hispanic	133	23.89 (9.07)	133	11.44 (5.89)	74	9.61 (4.55)
Spanish; Hispanic	4	25.81 (14.21)	4	13.04 (4.38)	2	6.85 (3.85)
Undocumented	1	34.26 (nan)	1	18.33 (nan)	1	13.06 (nan)
**Cancer Tissue**						
Breast	138	24.02 (9.19)	138	11.54 (5.86)	77	9.58 (4.52)
**Stage**						
I	55	21.86 (7.86)	54	11.11 (5.67)	25	9.38 (3.17)
II	50	24.96 (10.70)	51	10.56 (5.15)	28	10.09 (6.19)
III	22	28.39 (8.78)	22	13.37 (6.81)	13	9.25 (3.29)
IV	11	21.82 (4.85)	11	14.53 (6.80)	11	9.12 (3.73)
**T**						
T1	51	23.85 (9.36)	50	11.69 (5.48)	24	9.50 (3.19)
T2	43	23.86 (9.51)	43	11.83 (6.56)	26	9.59 (5.85)
T3	9	26.70 (4.86)	10	12.36 (5.69)	7	9.01 (2.49)
T4	5	26.79 (13.75)	5	13.14 (2.84)	4	9.17 (3.99)
U	30	23.29 (8.97)	30	10.34 (5.95)	16	10.03 (5.00)
**N**						
N0	67	22.86 (8.66)	67	12.06 (5.51)	36	9.74 (5.26)
N1	23	25.69 (10.32)	23	9.58 (4.44)	11	8.25 (2.20)
N2	10	32.27 (9.59)	10	15.77 (9.08)	6	10.71 (3.88)
N3	5	22.45 (3.17)	5	11.97 (5.02)	5	10.33 (1.60)
U	33	22.96 (8.90)	33	10.51 (5.84)	19	9.49 (4.86)
**M**						
M0	55	23.42 (7.35)	55	11.70 (5.13)	27	9.30 (3.09)
M1	5	20.29 (3.56)	5	11.90 (5.03)	5	8.55 (3.07)
U	78	24.69 (10.50)	78	11.41 (6.42)	45	9.86 (5.35)
**Specimen Considered**						
Benign	6	24.45 (4.30)	6	9.12 (3.30)	3	8.72 (0.11)
Metastatic	14	24.63 (9.57)	14	14.04 (9.50)	12	10.26 (4.49)
Primary	107	24.30 (9.61)	107	11.60 (5.34)	57	9.47 (4.74)
Recurrence	10	20.94 (5.70)	10	9.56 (5.06)	5	9.71 (4.17)
Undocumented	1	13.92 (nan)	1	4.13 (nan)	0	nan (nan)
**Tissue Site**						
Axillary	1	23.74 (nan)	1	7.12 (nan)	1	9.09 (nan)

Brain	4	28.65 (12.83)	4	23.13 (13.93)	4	11.47 (5.01)
Breast	118	24.03 (9.30)	118	11.33 (5.28)	63	9.45 (4.61)
Chest Wall	2	17.52 (3.22)	2	5.86 (4.56)	1	8.03 (nan)
Liver	2	25.86 (7.91)	2	13.17 (2.62)	2	8.38 (3.82)
Lung	1	30.22 (nan)	1	3.78 (nan)	0	nan (nan)
Lymph Node	5	21.70 (6.32)	5	13.57 (3.42)	3	11.08 (2.15)
Neck	1	40.82 (nan)	1	9.71 (nan)	1	18.20 (nan)
Ovary	2	17.38 (0.63)	2	10.17 (0.16)	2	5.44 (0.96)
Undocumented	2	20.14 (8.80)	2	7.85 (5.25)	0	nan (nan)
**Smoking History**						
Current smoker	15	26.55 (9.49)	15	11.61 (3.26)	7	9.78 (4.86)
Never smoked	75	23.53 (9.21)	76	11.79 (6.52)	46	10.03 (5.28)
Previous smoker	41	24.24 (9.11)	40	11.53 (5.38)	23	8.75 (2.37)
Undocumented	7	22.63 (9.89)	7	8.76 (5.32)	1	6.41 (nan)
**Tissue Exposure**						
Yes	42	26.27 (9.63)	43	9.81 (3.69)	17	10.06 (4.35)
No	33	21.95 (10.40)	33	9.00 (3.77)	14	11.14 (7.31)
Undocumented	63	23.61 (7.98)	62	14.09 (6.92)	46	8.93 (3.33)
**IHC Assay ER**						
Positive	95	24.38 (8.61)	94	11.59 (5.36)	49	8.82 (2.90)
Negative	43	23.24 (10.42)	44	11.43 (6.86)	28	10.91 (6.31)
**IHC Assay PR**						
Positive	80	24.18 (8.64)	79	12.09 (5.92)	45	8.74 (2.83)
Negative	58	23.81 (9.97)	59	10.81 (5.73)	32	10.75 (6.03)
**IHC Assay HER-2**						
Amplified	49	25.21 (10.94)	48	11.03 (6.02)	27	11.03 (6.06)
Not Amplified	89	23.37 (8.07)	90	11.81 (5.78)	50	8.79 (3.23)
**Subtype**						
Luminal A	79	24.74 (8.73)	78	12.01 (5.52)	41	9.01 (2.84)
Luminal B	18	22.66 (7.58)	18	10.92 (6.41)	10	8.80 (3.48)
HER2 Enriched	10	27.04 (14.97)	10	13.09 (9.82)	10	13.54 (9.15)
Triple Negative	31	22.01 (8.86)	32	10.25 (4.69)	16	9.04 (3.55)

U, Undocumented; nan, not a number
